# Idiopathic Spontaneous Intraperitoneal Hemorrhage Due to Vascular Malformations in the Muscularis of the Stomach: A Case Report

**DOI:** 10.3389/fmed.2022.927899

**Published:** 2022-09-01

**Authors:** Yuhang Zhou, Yuchen Zhou, Weihua Li, Shengtao Lin

**Affiliations:** ^1^Shengli Clinical Medical College of Fujian Medical University, Fuzhou, China; ^2^Department of Surgical Oncology, Fujian Provincial Hospital, Fuzhou, China; ^3^The First Affiliated Hospital of Fujian Medical University, Fuzhou, China

**Keywords:** idiopathic spontaneous intraperitoneal hemorrhage, abdominal apoplexy, vascular malformation, endoscopic ultrasonography, case report

## Abstract

Idiopathic spontaneous intraperitoneal hemorrhage (ISIH) is a phenomenon caused by spontaneous rupture of intra-abdominal visceral vessels, and vascular malformations (VMs) leading to ISIH are rare in previously reported cases. VMs of the gastric wall, which are commonly located in the mucosa and submucosa, mostly lead to upper gastrointestinal bleeding rather than intraperitoneal hemorrhage. To our knowledge, this is the first report of ISIH caused by VMs in gastric muscularis. In the current case, a 22-year-old male patient presented with sudden abdominal pain for 4 h, accompanied by tachycardia and hypotension. CT revealed a hematoma in the omental bursa and fluids in abdominopelvic cavities. Then intraperitoneal hemorrhage was confirmed after abdominal paracentesis. Furthermore, ultrasonic gastroscopy indicated that vascular malformation in the muscularis of the stomach probably led to intraperitoneal hemorrhage. The patient recovered after conservative treatment based on fluid resuscitation and remained stable for 12 months of follow-up. This case suggests that VMs located in the gastrointestinal tract may lead to ISIH and ultrasonic gastroscopy is helpful in the diagnosis of VMs in the gastrointestinal tract.

## Introduction

Idiopathic spontaneous intraperitoneal hemorrhage (ISIH), which was once labeled abdominal apoplexy, is a rare and potentially fatal condition caused by a spontaneous tear of intraperitoneal visceral vessels (1, 2). The presentation varies from non-specific abdominal pain to hemodynamic instability depending on the location and severity of bleeding (3). Surgical exploration remains a major diagnostic and therapeutic modality (4). In previously reported cases, ISIHs were commonly caused by atherosclerosis, aneurysms, vasculitis, etc. (2, 4). ISIH caused by vascular malformations (VMs) is rarely seen (5). VMs are congenital anomalies that can affect each part of the vasculature (6). About 90% of gastrointestinal VMs occur in the small intestine, leading to gastrointestinal bleeding (7, 8). To our knowledge, this is the first case of ISIH due to VMs in the muscularis of the stomach, which has some guidance for the etiology, diagnosis, and treatment of ISIH.

## Case Presentation

A 22-year-old Asian man was admitted with “severe mid-upper abdomen pain for 4 h.” He presented persistent, severe pain in the upper abdomen and pain radiating to the left shoulder, accompanied by dizziness, palpitations, amaurosis, and cold sweats. He did not receive any treatment before the admission and denied surgery, trauma, alcohol intake, strenuous exercise, and use of non-steroidal anti-inflammatory drugs before the onset. No family history of related illness was reported. On physical examination, he was noted to have tachycardia (heart rate: 106 beats per min) and hypotension (blood pressure: 81/50 mmHg). Mild abdominal distension, epigastric tenderness, and rebound pain were also noted during physical examination.

**FIGURE 1:**
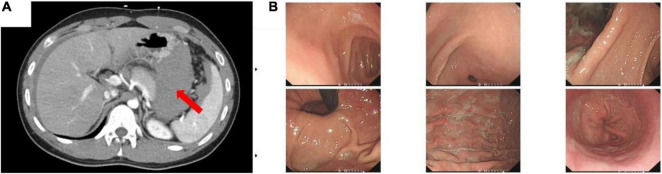
**(A)** Enhanced computed tomography (CT) of the abdomen. Red arrow showed a 10 × 5.2 cm^2^ high-density shadow in the gastro-pancreatic gap. **(B)** Gastroscopy showed that the gastric mucosa was smooth and had no ulcer or bleeding spot in the upper digestive tract.

Initial laboratory evaluation revealed a white cell count (WBC) of 16.82 × 10^9^/L, hemoglobin (HGB) of 15 g/dL, and hematocrit (HCT) of 46.3% (Table 1). Abdominal enhanced computed tomography (CT) showed a 10 × 5.2 cm^2^ hemoperitoneum in the gastro-pancreatic gap in addition to the massive hemoperitoneum around the spleen and in the rectovesical space, which was connected with the hemoperitoneum around the liver through the Winslow foramen (Figure 1A); and the size and density of each solid organ were normal. Subsequently, the patient underwent diagnostic abdominal paracentesis, which further confirmed that the intraperitoneal fluid was blood. Gastroscopy showed that the gastric mucosa was smooth, and no ulcer or bleeding spot was observed in the upper digestive tract (Figure 1B). Fluid resuscitation was conducted immediately after admission. Although blood routine tests revealed a downward trend of hemoglobin and hematocrit (HGB 13.1 g/dL, HCT of 37.8%), the patient’s condition tended to be stabilized (heart rate: 88 beats per min; blood pressure: 130/75 mmHg). Given the success of rehydration therapy and ambiguous source of hemorrhage, conservative medical treatment (Fluid resuscitation, Octreotide Acetate, Pantoprazole, etc.) was initially adopted. Meanwhile, the patient was under close observation, and emergency surgical exploration would be conducted if necessary.

**TABLE 1:** Labs during hospitalization.

Laboratory test (Normal range)	Value^1^	Value^2^	Value^3^	Value^4^	Value^5^
WBC (3.5–9.5 × 10^9^/L)	16.82	12.18	10.24	8.57	7.64
RBC (4.3–5.8 × 10^12^/L)	4.73	4.14	3.87	3.52	3.76
HGB (13.0–17.5 g/dL)	15.0	13.1	12.2	11.1	11.9
HCT (40.0–50.0%)	46.3%	37.8%	34.9%	31.6%	33.9%

*WBC, white blood cells; RBC, red blood cells; HGB, hemoglobin; PLT, platelet; HCT, hematocrit; Value^1^: Initial labs on admission; Value^2,3,4^: Labs of decompensation period every 5 h after expanding blood volume; Value^5^: Final labs at discharge.*

**FIGURE 2:**
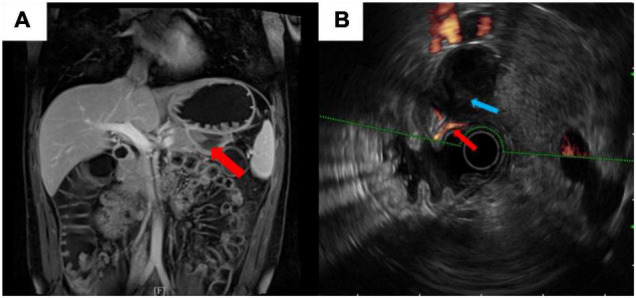
**(A)** Enhanced magnetic resonance imaging (MRI) revealed no sign of space-occupying lesion. Red arrow showed a hematoma in the gastro-pancreatic gap. **(B)** Endoscopic ultrasonography (EUS) illustrated the vascular malformation in the muscularis of the stomach. Blue arrow showed a hypoechoic shadow. Red arrow showed a 1.3-mm diameter blood flow signal was continuous between the muscularis propria and the hypoechoic shadow.

**FIGURE 3:**
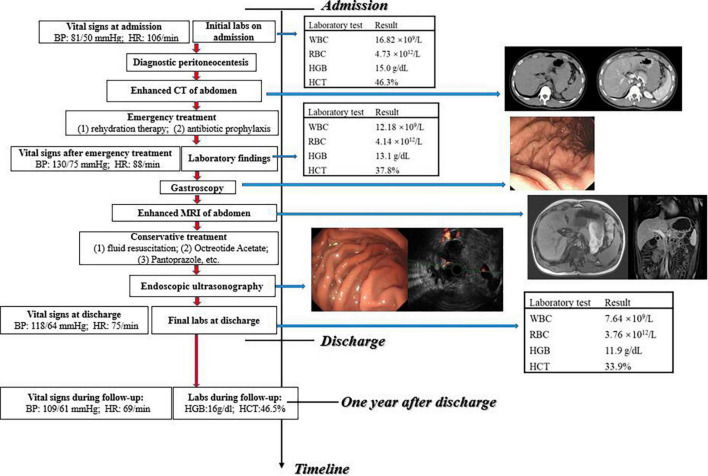
Timeline from emergency to follow-up.

To clarify the reason for the hemorrhage, enhanced magnetic resonance imaging (MRI) was performed, which revealed a hematoma in the gastro-pancreatic gap, without signs of space-occupying lesion and other indications about the source of bleeding (Figure 2A). Later, we performed endoscopic ultrasonography (EUS) on him. A mixed hypoechoic and anechoic lesion was seen between the pancreatic tail and the gastric wall, which was closely related to the peripheral blood vessels and was considered to be a hematoma combined with the previous imaging reports. Part of the muscularis propria was connected to the hematoma. In addition, Doppler ultrasonography showed that the blood flow signal was continuous between the muscularis propria and hematoma, which was considered as the vascular malformation in the muscularis of the stomach (Figure 2B). He was ultimately diagnosed with “intraperitoneal hemorrhage due to VMs in the muscularis of the stomach” based on the CT images and the EUS results, combined with the clinical manifestation. The patient was young and had fertility demands shortly. He was reluctant to accept CTA and interventional therapy due to the radiation dose of CTA and endovascular treatment. Also, considering that no obvious bleeding site was found on enhanced CT, we were concerned that the intervention would not be able to locate the lesion, so we did not conduct invasive treatment, such as endovascular management or surgery. The patient recovered gradually after conservative treatment without signs of continuous bleeding, and he was discharged after 4 days (the final laboratory data are shown in Table 1). We performed follow-ups for the patient at the hospital outpatient department at 6 months intervals. The follow-up after 1 year showed that the patient had no signs of recurrence (Figure 3). The timeline from emergency to follow-up is presented in Figure 3.

## Discussion

Idiopathic spontaneous intraperitoneal hemorrhage (ISIH), originally known as abdominal apoplexy, is used to describe atypical and non-traumatic spontaneous intraperitoneal or retroperitoneal bleeding, excluding typical intraperitoneal bleeding caused by ectopic pregnancy, malignant tumor, aortic aneurysm or dissection, visceral rupture, trauma, and iatrogenic injury (9, 10). Abdominal aneurysm is the main reason of ISIH, of which splenic aneurysm is the most common (60%) and the gastric and gastroepiploic aneurysms are responsible for only 3% (2). The rupture of a splenic aneurysm is mostly caused by the erosion of the vessel wall owing to pancreatic enzymes released in pancreatitis (in about 50% of cases) and results in over 90% mortality (11). Besides abdominal aneurysms, atherosclerosis is considered to be another important cause of ISIH. The presumed mechanism is the weakness of the tunica media in the injured vessel, which ruptures when blood pressure suddenly rises (2). As described in the report, a patient was presented to our hospital with sudden, non-specific abdominal pain and, was finally diagnosed with ISIH caused by VMs in gastric muscularis mainly relying on EUS.

VMs are diseases with unpredictable clinical evolution and manifestations, which lead to life-threatening conditions in severe cases (12). The splenic artery is the most common vessel responsible for ISIH, while the gastric or gastroepiploic artery accounts for only 4% (13). The VMs of the gastric wall generally occur in the gastric mucosa and submucosa, so it usually causes intragastric hemorrhage (14–16). We checked the relevant literature and found that this is the first case of a vessel in the muscularis of the stomach inducing intraperitoneal bleeding, which suggests that the blood vessels of the gastric serosal surface, and even the entire stomach wall, should be considered when looking for the bleeding sites of ISIH.

According to the available literature, optimal diagnostic and therapeutic evidence of ISIH remain controversial (12). CT angiography (CTA) or digital subtraction angiography (DSA) is considered to be a primary tool for diagnosing ISIH, localizing the bleeding vessel if the hemodynamic and clinical status of patients enables it (3, 17). The non-invasiveness and short acquisition time of CTA give it more advantages for acute bleeding and a higher priority than DSA (18, 19). However, some literature points out that CTA may have false-negative results which can occur if the bleeding is not obvious at the time of the scan due to the short acquisition time (19–21). Considering that the patient had no indication of active bleeding, and it was difficult to locate the culprit vessel using CTA, we did not perform CTA in this case. EUS can provide real-time images of the gastrointestinal wall and blood vessels of adjacent tissues and has been applied to diagnostic interventions (22, 23). VMs can appear as a persistent blood flow signal in the parietal layer of the gastrointestinal tract under EUS guidance (24).

Surgery and embolization are methods for the treatment of VMs. Endovascular embolization is less invasive and recommended in most cases (9). However, it is difficult to locate culprit vessels in cases without active bleeding, so endovascular embolization was not selected in this case. Exploratory surgery is usually employed when CTA cannot be performed or failed to identify the culprit vessel (17). Exploratory laparotomy or laparoscopic exploration can detect the bleeding site, achieve initial hemostasis (25), and take tissue for pathological diagnosis. However, nearly 40% of operations failed in finding the bleeding site of the patient (9, 26). Therefore, due to the lack of continuous bleeding, we did not surgically explore and treat the patient. For younger patients without immediate surgical management, we suggest close attention and a conservative administration, including somatostatin or somatostatin analogs (such as octreotide), which have been concluded as an effective therapy for hemorrhage of gastrointestinal VMs (27, 28). However, for patients with decompensated or life-threatening conditions, we recommend emergency surgery; for patients with recurrent bleeding, surgery may be also finally required (29).

The limitations of our study include the lack of objectivity of imaging and the ambiguous assessment of therapeutic effect. Different from CT or MRI, EUS is operator dependent, and the analysis of the images is subjective. There is a risk of recurrence because the patient did not receive surgical treatment and EUS revealed that VMs still existed, though he is currently in stable condition. The length of follow-up time is not sufficient to accurately evaluate the effect of conservative treatment up to now.

## Conclusion

For patients with spontaneous intraperitoneal hemorrhage, particularly ISIH, VM rupture should be considered when the disease cannot be identified after excluding the common causes. VMs in the gastric wall may lead to intraperitoneal hemorrhage in addition to intragastric bleeding. If it is speculated that the vascular malformation is located in the gastric wall, EUS may be a new alternative diagnostic approach for CTA. Due to the lack of evidence, it is difficult to standardize the treatment of such patients. Conservative treatment can be chosen temporarily for patients without immediate surgery or indication of embolism.

## Data Availability Statement

The original contributions presented in this study are included in the article/supplementary material, further inquiries can be directed to the corresponding author/s.

## Ethics Statement

Written informed consent was obtained from the individual(s) for the publication of any potentially identifiable images or data included in this article.

## Author Contributions

YHZ, YCZ, WL, and SL participated in the diagnosis and treatment of patients. YHZ and YCZ wrote the manuscript. WL provided professional opinions on diagnosis. SL reviewed and revised the manuscript. All authors contributed to manuscript revision, read, and approved the submitted version.

## Conflict of Interest

The authors declare that the research was conducted in the absence of any commercial or financial relationships that could be construed as a potential conflict of interest.

## Publisher’s Note

All claims expressed in this article are solely those of the authors and do not necessarily represent those of their affiliated organizations, or those of the publisher, the editors and the reviewers. Any product that may be evaluated in this article, or claim that may be made by its manufacturer, is not guaranteed or endorsed by the publisher.
